# Supplementary material for: A Novel Murine Multi-Hit Model of Perinatal Acute Diffuse White Matter Injury Recapitulates Major Features of Human Disease
Source: Biomedicines. 2022 Nov 4;10(11):2810. doi: 10.3390/biomedicines10112810 (PMC9687579; doi:10.3390/biomedicines10112810)
Supplement: Supplementary file 1 [file biomedicines-10-02810-s001.zip › Supplementary Figure S1.pdf]

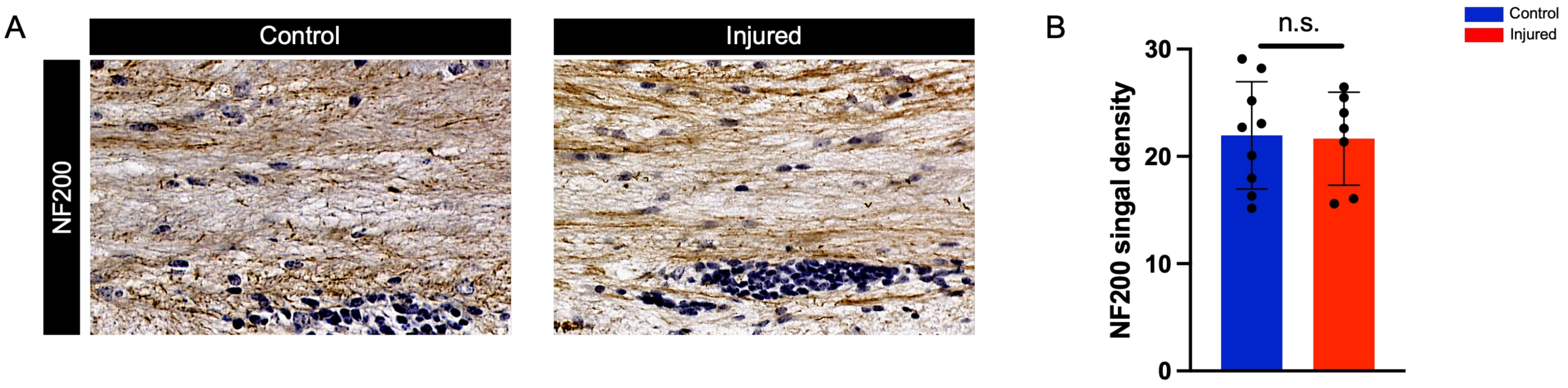

Supplementary Figure S1: Evaluation of neurofilament density. (A) Representative images of NF200 staining of a control and injured brain at 9dpi. (B) Quantification of NF200 signal density in the corpus callosum ($U = 30$, $p = 0.9182$). Data are presented as mean ± SEM. n.s. not significant.
