# Supplementary material for: A Novel Murine Multi-Hit Model of Perinatal Acute Diffuse White Matter Injury Recapitulates Major Features of Human Disease
Source: Biomedicines. 2022 Nov 4;10(11):2810. doi: 10.3390/biomedicines10112810 (PMC9687579; doi:10.3390/biomedicines10112810)
Supplement: Supplementary file 1 [file biomedicines-10-02810-s001.zip › Supplementary Table S1.pdf]

Supplementary Table S1: Summary of experimental cohorts

Mouse Nr	Experiment	Age at euthanazation	Treatment		Notes	Corpus callosum							Cortex				Internal capsule/ Golubs pallidus		
						Olig2+ Ki67+	Olig2+ TUNEL	Olig2+ CNPase +	Ng2	GFAP	Iba1	MBP	GFAP	Iba1	MBP	TUNEL	GFAP	Iba1	MBP
**M83**	16	P11	2 mg/kg NaCl	Normoxia 25 MIN		x	x	x		x	x	x	x	x	x	x	x	x	x
**M84**	16	P11	2 mg/kg NaCl	Normoxia 25 MIN		x	x	x		x	x	x	x	x	x	x	x	x	x
**M85**	16	P11	2 mg/kg NaCl	Normoxia 25 MIN		x	x	x		x	x	x	x	x	x	x	x	x	x
**M86**	16	P11	2 mg/kg LPS	25 MIN @ 8% O2		x	x	x		x	x	x	x	x	x	x	x	x	x
**M87**	16	P11	2 mg/kg LPS	25 MIN @ 8% O2		x	x	x		x	x	x	x	x	x	x	x	x	x
**M88**	16	P11	2 mg/kg LPS	25 MIN @ 8% O2		x	x	x		x	x	x	x	x	x	x	x	x	x
**M99**	16	P11	2 mg/kg NaCl	Normoxia 25 MIN		x	x	x		x	x	x	x	x	x	x	x	x	x
**M100**	16	P11	2 mg/kg NaCl	Normoxia 25 MIN		x	x	x		x	x	x	x	x	x	x	x	x	x
**M101**	16	P11	2 mg/kg NaCl	Normoxia 25 MIN		x	x	x		x	x	x	x	x	x	x	x	x	x
**M115**	20	P11	2 mg/kg NaCl	Normoxia 25 MIN	Tissue not analyzed; tissue integrity compromised														
**M116**	20	P11	2 mg/kg NaCl	Normoxia 25 MIN	Tissue not analyzed for MBP; Scanning failure	x	x	x		x	x		x	x		x	x	x	
**M117**	20	P11	2 mg/kg LPS	25 MIN @ 8% O2	Tissue not analyzed for MBP & TUNEL; Scanning failure	x		x		x	x		x	x		x	x	x	
**M118**	20	P11	2 mg/kg LPS	25 MIN @ 8% O2	Tissue not analyzed for MBP; Scanning failure	x	x	x		x	x		x	x		x	x	x	
**M119**	21	P11	2 mg/kg NaCl	Normoxia 25 MIN		x	x	x		x	x	x	x	x	x	x	x	x	x
**M120**	21	P11	2 mg/kg NaCl	Normoxia 25 MIN	Tissue not analyzed; plane inappropriate														
**M121**	21	P11	2 mg/kg LPS	25 MIN @ 8% O2	Tissue not analyzed for MBP; plane inappropriate	x	x	x		x	x	x	x	x	x	x	x	x	
**M122**	21	P11	2 mg/kg LPS	25 MIN @ 8% O2		x	x	x		x	x	x	x	x	x	x	x	x	x
**M123**	21	P11	2 mg/kg LPS	25 MIN @ 8% O2		x	x	x	x	x	x	x	x	x	x	x	x	x	x
**M135**	23	P3	2 mg/kg NaCl	Normoxia 25 MIN		x	x	x	x	x	x		x	x		x	x	x	
**M136**	23	P3	2 mg/kg NaCl	Normoxia 25 MIN		x	x	x	x	x	x		x	x		x	x	x	
**M137**	23	P3	2 mg/kg LPS	25 MIN @ 8% O2	Corpus callosum not analyzed for Iba1/GFAP due to inappropriate plane/ compromised anatomy	x	x	x	x							x	x	x	
**M138**	23	P3	2 mg/kg LPS	25 MIN @ 8% O2		x	x	x	x	x	x		x	x		x	x	x	
**M139**	23	P3	2 mg/kg LPS	25 MIN @ 8% O2		x	x	x	x	x	x		x	x		x	x	x	
**M140**	23	P4	2 mg/kg NaCl	Normoxia 25 MIN		x	x	x	x	x	x		x	x		x		x	
**M141**	23	P4	2 mg/kg NaCl	Normoxia 25 MIN		x	x	x	x	x	x		x	x		x		x	
**M142**	23	P4	2 mg/kg LPS	25 MIN @ 8% O2		x	x	x	x	x	x		x	x		x		x	
**M143**	23	P4	2 mg/kg LPS	25 MIN @ 8% O2		x	x	x	x	x	x		x	x		x		x	
**M144**	23	P11	2 mg/kg NaCl	Normoxia 25 MIN		x	x	x		x	x	x	x	x	x	x	x	x	x
**M145**	23	P11	2 mg/kg LPS	25 MIN @ 8% O2		x	x	x		x	x	x	x	x	x	x	x	x	x
**M146**	24	P3	2 mg/kg NaCl	Normoxia 25 MIN		x	x	x	x	x	x		x	x		x	x	x	
**M154**	24	P4	2 mg/kg NaCl	Normoxia 25 MIN		x	x	x	x	x	x		x	x		x	x	x	
**M155**	24	P4	2 mg/kg NaCl	Normoxia 25 MIN	Corpus callosum not analyzed for Iba1/GFAP due to inappropriate plane/ compromised anatomy	x	x	x	x				x	x		x		x	
**M156**	24	P4	2 mg/kg NaCl	Normoxia 25 MIN		x	x	x	x	x	x		x	x		x	x	x	
**M157**	24	P4	2 mg/kg LPS	25 MIN @ 8% O2	Corpus callosum not analyzed for Iba1/GFAP due to inappropriate plane/ compromised anatomy	x	x	x	x				x	x		x			
**M158**	24	P11	2 mg/kg NaCl	Normoxia 25 MIN		x	x	x		x	x	x	x	x	x	x	x	x	x
**M159**	24	P11	2 mg/kg NaCl	Normoxia 25 MIN		x	x	x		x	x	x	x	x	x	x	x	x	x
**M160**	25	P3	2 mg/kg NaCl	Normoxia 25 MIN		x	x	x	x		x		x	x		x	x	x	
**M161**	25	P3	2 mg/kg LPS	25 MIN @ 8% O2	Corpus callosum and cortex not analyzed for Iba1/GFAP due to inappropriate plane/ compromised anatomy	x	x	x	x				x	x		x	x	x	
**M162**	25	P3	2 mg/kg NaCl	Normoxia 25 MIN		x	x	x	x	x	x		x	x		x	x	x	
**M163**	25	P3	2 mg/kg LPS	25 MIN @ 8% O2	Tissue not analyzed; tissue integrity compromised														
**M165**	25	P3	2 mg/kg LPS	25 MIN @ 8% O2		x	x	x	x	x	x		x	x		x	x	x	
**M166**	25	P3	2 mg/kg LPS	25 MIN @ 8% O2		x	x	x	x	x	x		x	x		x	x	x	
**M167**	25	P4	2 mg/kg NaCl	Normoxia 25 MIN		x	x	x	x	x	x		x	x		x	x	x	
**M168**	25	P4	2 mg/kg LPS	25 MIN @ 8% O2		x	x	x	x	x	x		x	x		x	x	x	
**M169**	25	P4	2 mg/kg LPS	25 MIN @ 8% O2		x	x	x	x	x	x		x	x		x	x	x	
**M170**	25	P4	2 mg/kg LPS	25 MIN @ 8% O2		x	x	x	x	x			x	x		x	x	x	
**M171**	25	P4	2 mg/kg LPS	25 MIN @ 8% O2		x	x	x	x	x	x		x	x		x	x	x	
